# Transportation container for pre-processing cytogenetic assays in radiation accidents

**DOI:** 10.1038/s41598-021-89832-x

**Published:** 2021-05-17

**Authors:** Jian Gu, Brett Duane, Mikhail Repin, David J. Brenner, Frederic Zenhausern

**Affiliations:** 1 Center for Applied NanoBioscience and Medicine, Department of Basic Medical Sciences, The University of Arizona, College of Medicine, Phoenix, AZ 85004 USA; 2 Center for Radiological Research, Columbia University, Vagelos College of Physicians and Surgeons, New York, NY 10032 USA

**Keywords:** Public health, Population screening, Biomedical engineering

## Abstract

We report a shipping container that enables a disruptive logistics for cytogenetic biodosimetry for radiation countermeasures through pre-processing cell culture during transportation. The container showed precise temperature control (< 0.01 °C) with uniform sample temperature (< 0.1 °C) to meet the biodosimetry assay requirements. Using an existing insulated shipping box and long shelf life alkaline batteries makes it ideal for national stockpile. Dose curve of cytogenetic biodosimetry assay using the shipping container showed clear dose response and high linear correlation with the control dose curve using a laboratory incubator (Pearson’s correlation coefficient: 0.992). The container’s ability of pre-processing biological samples during transportation could have a significant impact on radiation countermeasure, as well as potential impacts in other applications such as biobanking, novel molecular or cell-based assays or therapies.

## Introduction

Exposure to ionizing radiation continues to be a threat to public safety should a nuclear/radiological event happen, such as an improvised nuclear device (IND) or a radiation dispersal device (RDD) detonated by a terrorist, or a nuclear power plant disaster caused by nature or human error^1,2^. The dangerous exposure zone can cover a large area due to different spreading routes of the radioactive contaminants, which can cause a large population to worry about their exposure level. In this scenario, a rapid triage tool is critical to give patients needed treatment as well as to ease the anxiety of the “healthy worries”^3^.

Triage after a nuclear/radiological event can be multiple stages by different dosimetry assays^3^. After an initial point-of-care (POC) triage, a high-throughput dosimetry system is expected to give more accurate dose estimates. Sullivan et al. assessed different biodosimetry methods for radiation countermeasure, including lymphocyte depletion kinetics (LDK) assay, γ-H2AX assay, dicentric chromosome assay (DCA), cytokinesis-block micronucleus (CBMN) assay etc^3^. These assays differ by their complexity, applicable ranges of time after the radiological event and dose. For example, the LDK assay can be performed in a POC setting, and a rough dose estimate could be obtained by a single cell count. The γ-H2AX assay has a dose range of 0.5–5 Gy and time range of 24–48 h, which could be suitable for a quick initial triage, but its short time window could also limit its usability. Cytogenetic assays, such as DCA and CBMN, are more established assays, and DCA is considered the “gold standard” for biodosimetry.

Among the different biodosimetry assays, CBMN is one of the cytogenetic biodosimetry assays recommended by the International Atomic Energy Agency (IAEA) for radiation exposure by examining micronucleus formation from human peripheral blood lymphocytes^4^. It is promising for large population triage application because it is an established assay that could be deployed quickly, and it is more suitable for automation comparing with other established cytogenetic biodosimetry assays (such as DCA). Recently, automation of the CBMN assay has been demonstrated for high-throughput processing of large number of samples using commercial robotic systems^5,6^. To streamline the dosimetry process in the aftermath of a nuclear/radiological event, a fingerstick blood self-collector was also developed to address the sample collection bottleneck^7^. Despite these efforts, however, challenges remain to apply CBMN for population triage after a large scale nuclear/radiological event.

**Figure 1:**
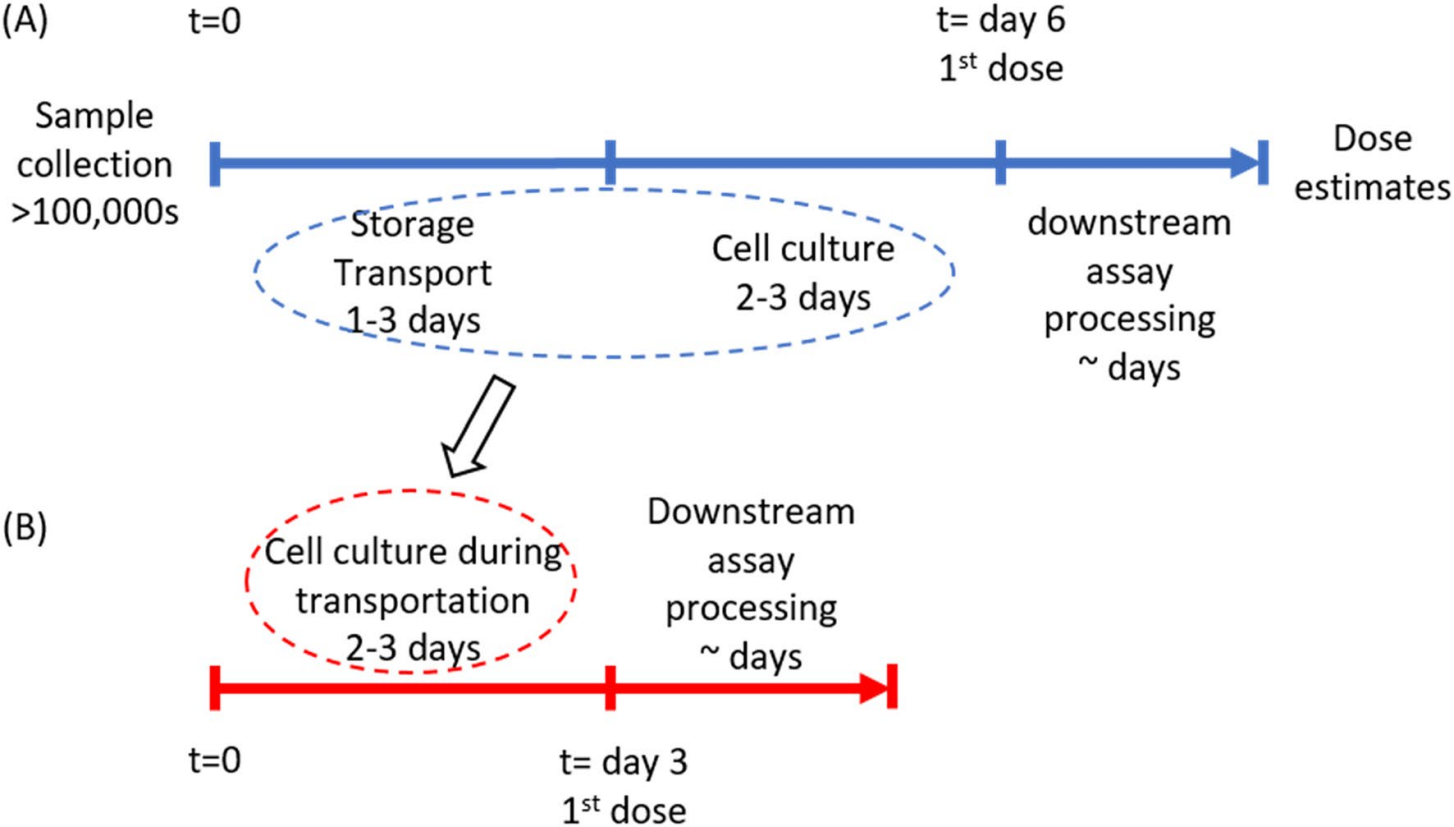
(**A**) Existing cytogenetic biodosimetry logistics: the response time to get the 1st dose result can be as long as 6 days due to shipping and cell culture; (**B**) our novel cytogenetic biodosimetry logistics: cell culture during transportation can dramatically decrease the response time of the biodosimetry to 3 days.

The main challenge of CBMN for radiation triage is its long response time due to sample transportation and a required cell culture step at beginning of the assay. Figure 1A shows the current logistics of CBMN assay for radiation triage. Due to the complexity of the CBMN assay, samples must be shipped to a central laboratory for processing after collection. With limited cytogenetic laboratories within the USA and around the world, air transportation is expected. The shipping time can last 3 days or longer depending on weather conditions, possible aircraft mechanical issues and delays at customs. Once samples are received, blood cells need to be cultured for ~ 72 h before they can be harvested and micronuclei are identified. Even though the cell culture time can be shortened to 54 h using an accelerated CBMN assay^8^, the overall response time (i.e., the time for the first dose estimate to be available after sample collection) can still be 6 days or longer. To address this long response time, we propose a novel logistics for the CBMN assay, i.e., to use a shipping incubator to perform the cell culture immediately after the sample collection and during transportation (Fig. 1B). In this way, the assay response time can be dramatically reduced to ~ 3 days, half of the current response time.

There are multiple requirements for a shipping container to perform cell culture for CBMN assay for radiation countermeasure. First, to ensure accurate cytogenetic endpoints are detected, the temperature of the cell culture needs to be controlled within 0.5 °C of 37 °C^4,9^. Second, the battery needs to last for the entire culture duration (54–72 h) even in cold winter weather. Third, it needs to be low cost and easy to store in the national stockpile. There are previous reports on temperature-controlled shipment of biological samples^10,11^, but the temperature usually spans a range of several degrees Celsius, too wide for CBMN assay. There are also commercial shipping incubators (e.g., iQ from MicroQ Technologies, Scottsdale, AZ, or BioTherm from CryoLogic, Australia) that can control the shipping temperature within 0.5 °C of 37 °C, but they are usually bulky, high cost with rechargeable batteries (that requires charging before use and may fail in storage) and not suitable for national stockpile. In this paper, we report our efforts to build a shipping container for CBMN cell culture. We call it micro-Cytogenetic Rapid Assay Integrated Transporter (µCRAIT) box. The name also contains all the key elements of the container, i.e., micro-Controlled Resistive heating of long shelf life Alkaline-battery-powered traditional Insulating shipping box for precise Temperature control. The box design, temperature control, battery life, resistance to mechanical shock, and initial application to CBMN assay with cell culture during transportation will be reported.

## Experimental results

### µCRAIT box design and prototyping

**Figure 2:**
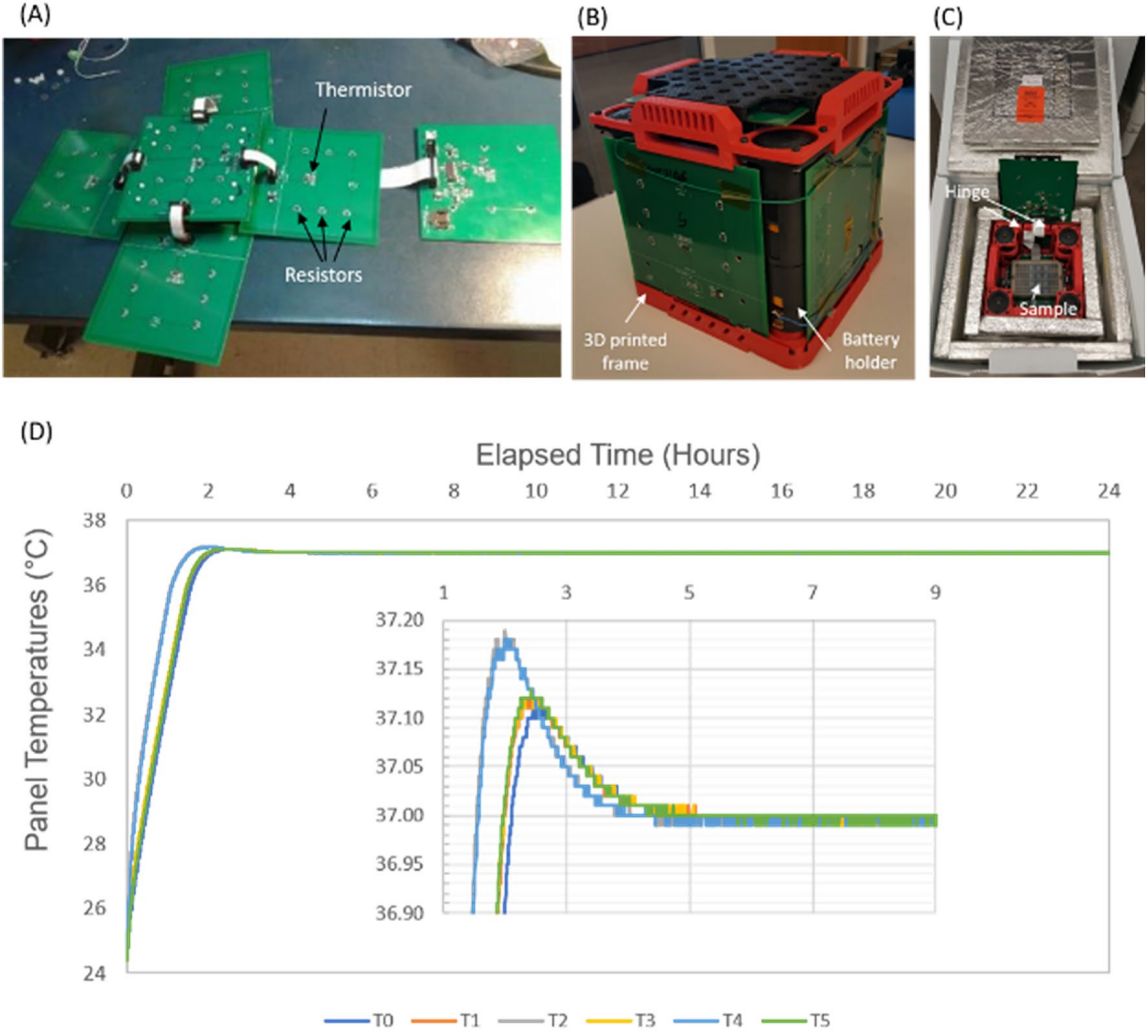
(**A**) Six PCBs connected by ribbon cables that are used to form the temperature-controlled isothermal internal surfaces of a µCRAIT box. Each PCB temperature is controlled by resistive heating of six resistors and microcontroller feedback of a thermistor; (**B**) a 3D-printed frame that holds the six PCBs, as well as 4 battery holders at the corners; (**C**) a µCRAIT box by housing the 3D-printed PCB frame box inside a traditional dual-wall VIP insulated shipping box; (**D**) typical temperature curves of the six PCB panels heated towards 37 °C from room temperature.

The main concept of the µCRAIT box starts with the idea that the internal box temperature can be controlled by making the six box internal surfaces an isothermal surface, which will make the box temperature the same as the isothermal surface temperature. To control the six internal surfaces at the desired 37 °C, a printed circuit board (PCB) is used for each surface as an individual temperature-controlled surface (Fig. 2A). The PCB not only supports the temperature control circuits, but also serves as a good heat conductor for uniform temperature across the board due to internal copper layers. The temperature of each PCB is measured by a thermistor mounted on the board. Six resistors distributed across the board are used to maintain the board temperature at 37 °C by resistive heating (Fig. 2A). A 3D printed frame is used to mechanically hold the six PCBs together (Fig. 2B). It also holds four battery holders at the corners. Then the system can be loaded into a traditional insulating shipping box to generate the 37 °C environment for cell culture during transportation (Fig. 2C). Two commercial vacuum-insulated-panel (VIP) shipping boxes were used in our study due to the low thermal conductivity of the VIP material, a dual-wall box and a single-wall box. The top of the frame with the PCB can be flipped open with a printed hinge for sample loading.

To control the temperature, a microcontroller system is designed and implemented using the PCBs. The system is powered by 8 D-cell alkaline batteries for heating and operation (two for each battery holder). Alkaline battery is chosen due to its low cost and long shelf life (5–10 years), which is ideal for national stockpile. A coin cell (CR2032), which also has long shelf life (10–12 years), is used as a backup power for general operation (excluding heating) in case the event a transient impact causes the D cells to be momentarily disconnected. The system uses a proportional-integral-derivative (PID) feedback loop to accurately control the PCB temperature, and pulse-width-modulation (PWM) for heater power control. The PCB temperature is measured by resistance change of the thermistor in a Wheatstone bridge. The microcontroller also has the functions of real time clock (RTC), communication with external computer, and memory for data logging. The logged parameters include date and time, PCB temperatures, battery voltage, heating power and other significant events such as power disruption, disabling of heating etc. For our µCRAIT box using the dual-wall VIP shipping box, it usually takes about 2 h for the box to heat up from room temperature to 37 °C. Figure 2D shows a typical set of temperature curves of all six PCB panels heated toward 37 °C. The PID and PWM circuits accurately controlled the temperature at the setpoint within 0.01 °C.

### Temperature measurement, calibration and characterization

**Figure 3:**
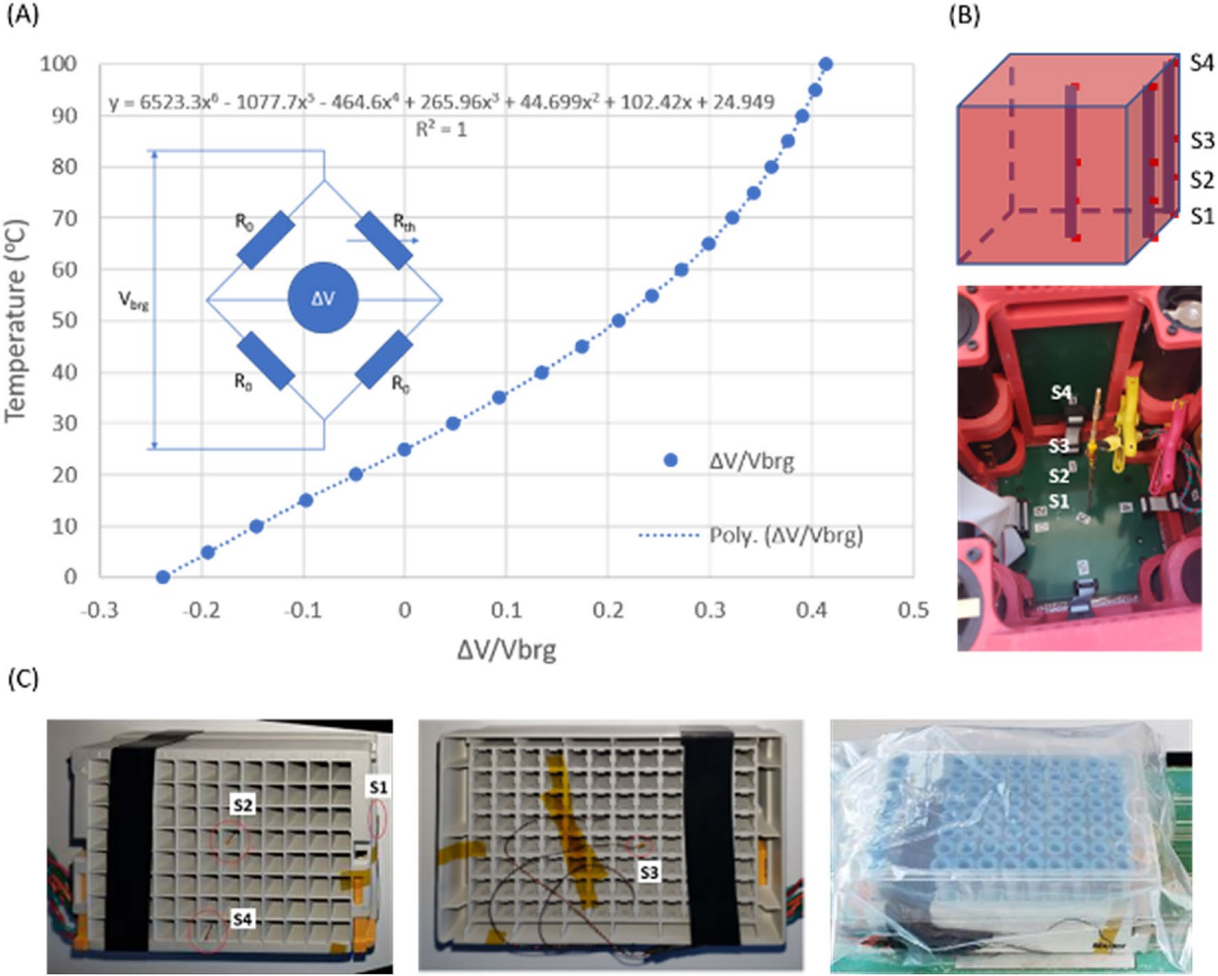
(**A**) Schematics of the Wheatstone bridge circuit; the T versus ∆V/Vbrg curve calculated using Eq. (1) and manufacturer’s thermistor resistance data was fitted by a sextic polynomial function with a R-Square value of 1; (**B**) schematics of the four wired thermistors on a stick to characterize the internal temperature of the µCRAIT box at the corner, side and center of the box; an image of the stick sensors measuring center temperature of the box is shown; (**C**) images showing the wired sensors to measure temperatures of the sample rack at different locations.

Precise temperature control ($37 \pm 0.5$ °C) is a main goal of the µCRAIT box. For accurate temperature measurement, a thermistor in a Wheatstone bridge (Fig. 3A) mounted on each PCB was used to measure the temperature of each board. A thermistor was chosen for its sensitivity, stability and ease for integration^12,13^. The Wheatstone bridge was chosen for its insensitivity to voltage variation. The temperature-dependent resistance of a thermistor can be described by the Steinhart–Hart equation^14^. However, it is more convenient for the microcontroller to compute temperature T directly from the unbalance of the bridge, i.e., the ratio between output and input voltages of the bridge ∆V/V_brg_. The unbalance of the bridge can be mathematically deduced as:

1 $$ \frac{\Delta V}{{V_{brg} }} = \frac{1}{2}*\frac{{R_{0} - R_{th} \left( T \right)}}{{R_{0} + R_{th} \left( T \right)}} $$

where R_0_ is the known resistance of the bridge resistors, R_th_ is the variable resistance of the thermistor. Using manufacturer’s R_th_(T) data, T versus ∆V/V_brg_ was plotted for the temperature range between 0 and 100 °C and fitted by a sextic polynomial function with a R^2^ (Goodness-of-Fit) value of 1 (Fig. 3A). The polynomial was then used by the microcontroller’s firmware to measure the PCB temperature.

For accurate temperature measurement, each temperature sensing circuit was calibrated using a Resistance Temperature Detector (RTD) reference thermometer. To do this, the µCRAIT box and the reference thermometer were put inside a laboratory incubator with a fan to reach uniform temperature inside the incubator. The PCB temperatures were read out at both 37 °C and 32 °C and compared with those from the reference thermometer. Because we are mainly concerned about the accuracy of the temperature at 37 °C, an offset was first used for each PCB to correct the temperature at 37 °C. The corrected temperature T_cor_ can be expressed as:

2 $$ T_{cor} = T_{raw} + Offset, \;Offset = T_{ref,37} - T_{raw,37} \quad so\;that\quad T_{cor,37} = T_{ref,37} $$

where T_raw_ is the raw PBC temperature measured by the unbalance of the Wheatstone bridge and the thermistor sextic polynomial curve, and T_ref,37_, T_raw,37_, T_cor,37_ are the reference, PBC, and offset-corrected temperature readings at the 37 °C calibration.

To further correct the temperature around 37 °C, because the T versus ∆V/V_brg_ curve around and below 37 °C is quite linear (Fig. 3A), a sloped linear correction was used for each PCB to make the board temperature the same as the RTD reading at 32 °C while keeping the 37 °C calibration intact. The further corrected temperature T_cor_′ for each PCB can be expressed as:

3 $$ T_{cor}^{\prime } = T_{cor} + Slope*\left( {T_{cor} - T_{cor,37} } \right),\;Slope = \frac{{T_{ref,32} - T_{cor,32} }}{{T_{cor,32} - T_{cor,37} }}\quad so\;that\quad T_{cor,32}^{\prime } = T_{ref,32} $$

where T_cor,32_ and T_ref,32_ are the offset-corrected PBC temperature and reference temperature readings at the 32 °C calibration.

We conducted six calibration experiments to collect information on the uncertainty of the temperature measurement. Table 1 lists the averages and standard deviations (STDs) of the Offsets and Slopes for all six PCBs from the six calibrations. We can see that the Offsets have average values of 0.407 to 1.145 °C with STD_Offset_ 0.015 to 0.037 °C. The Slopes vary between 0.0067 and 0.0106 with STD_Slope_ between 0.0018 and 0.0055. Because the box can control the temperature 0.01 °C or less away from the 37 °C setpoint, the Slope correction is not significant for the box temperature control at the setpoint. The low STD_Offset_ shows that the PBC can be quite accurately controlled at 37 °C with an error much smaller than our targeted 0.5 °C.

**Table 1:** Temperature characterization of the µCRAIT box and the wired thermistor sensors.

Sensor #	Offset (°C)	STD_offset_ (°C)	Slope	STD_Slope_
**µCRAIT PCBs**				
0	0.527	0.022	0.0084	0.0028
1	0.407	0.037	0.0067	0.0055
2	1.145	0.031	0.0106	0.0040
3	0.785	0.015	0.0089	0.0018
4	1.057	0.023	0.0088	0.0029
5	1.026	0.023	0.0087	0.0040
**Wired sensors**				
1	− 0.052	0.021	− 0.0287	0.0031
2	− 0.062	0.049	− 0.0327	0.0057
3	0.032	0.031	− 0.0304	0.0037
4	0.152	0.056	− 0.0234	0.0104

With the PCB temperature calibrated and heated to 37 °C, the internal temperature of the µCRAIT box was characterized by four wired thermistor sensors mounted to a stick with a length similar to the internal dimension of the box (Fig. 3B, sensors 1–4 were located at 0, 25, 50 and 100% positions of the stick from bottom to top). The wired thermistor temperature sensing circuits were constructed and calibrated similar to those for the PCB temperature sensing and calibration. The Offsets, Slopes, their STDs are also listed in Table 1. Because the sensors will measure temperatures very close to 37 °C (within 0.5 °C or less), the total STD can be estimated by STD_Offset_ + ∆T*STD_Slope_ with maximum values of 0.023–0.061 °C (∆T = 0.5 °C). The small STDs allow the sensors to characterize the box temperature within the targeted range (37 ± 0.5 °C).

With the sensors on the stick calibrated, the temperatures at corner, side and box center inside the µCRAIT box were measured when the box was heated to 37 °C (Fig. 3B). The box internal temperatures at the equilibrium are listed in Table 2. The values are calculated using Eq. (3) with a T_cor,37_ value of 36.93 °C. We can see that the spreading of the temperature for the whole box is less than 0.6 °C. It is more uniform in the center of the box (temperature spread < 0.12 °C) where the sample will be located. The corner and side of the box have slightly higher temperatures than the center because they are closer to the heat source. The top of the box (sensor 4) is also slightly warmer than the bottom (sensor 1) because warmer air has a lower density and tends to rise.

**Table 2:** µCRAIT box and sample rack temperature uniformity characterization.

Sensor#	1	2	3	4	Average	STD
**µCRAIT box**						
Corner	36.54	36.75	36.96	37.11	36.84	0.250
Side	36.57	36.69	36.78	36.85	36.72	0.120
Center	36.65	36.61	36.64	36.73	36.66	0.052
Dummy sample rack payload	36.71	36.64	36.65	36.73	36.68	0.046

For µCRAIT box, the sensors 1–4 were located at bottom, mid-bottom, middle and top of the stick.

For the dummy sample rack, the sensors 1–4 were located at outside short edge, inside center, outside bottom and inside long edge of the rack.

For CBMN assay, samples will be transported in 96-tube-rack format 2D-barcoded storage tubes. To further understand the temperature experienced by the samples, the wired sensors were used to measure the temperatures at different locations of a tube rack (Fig. 3C), loaded with dummy sample tubes and placed inside the center of the µCRAIT box. The steady state temperatures at different locations (S1: outer short side; S2: center; S3: bottom; S4: inner long side) are quite uniform across the sample rack with a temperature spread of 0.09 °C (Table 2). The average temperature is, however, 0.32 °C lower than 37 °C. We attribute this to the fact that our current PCB panels do not form a completely enclosed isothermal space, and heat can be lost through the space between the PCBs. To compensate this temperature drop, we raised the temperature setpoints of all PCBs to 37.3 °C for an average sample temperature of 36.98 °C for our CMBN assay blood culture during transportation. The overall uncertainty of the sample temperature can be estimated by the sum of maximum STDs from the PBCs (0.037 °C) and the wired sensors (0.061 °C), which shows a value of 0.098 °C. This low value puts the sample temperature well within the targeted 37 ± 0.5 °C range. We expect very low measurement variations at 32 °C and 37 °C from the RTD reference thermometer, which are neglected in our estimation. According to the manufacture specification, the RTD thermometer has an accuracy of ± 0.03 °C over the range of − 30 to 150 °C, which is low and will not affect our temperature measurement significantly.

### Battery selection and capacity

The battery is another key aspect of the µCRAIT box. The ideal battery should be low cost with long shelf life for national stockpile. It should also have enough capacity to power the box at 37 °C for several days during transportation, even in a cold winter.

Alkaline battery fits the national stockpile requirement very well due to its low cost and long shelf life (5–10 years), so it was selected as the main battery source for the system control and box heating. The common coin cell battery also has long shelf life (10–12 years) and was used as the backup power for the microcontroller operation excluding heating.

To maintain 37 °C during transportation, any heat loss due to lower ambient temperature needs to be compensated by the resistive heating of the PCBs. µCRAIT boxes use traditional insulating shipping boxes to reduce heat loss and extend the culture temperature duration. If we assume the heat loss is mainly due to thermal conductivity of the box’s insulation layer, we can use Fick’s Law to approximate the heat loss (dQ/dt) of the box with a simple 1D model:

4 $$ \frac{dQ}{{dt}} = - C*\frac{\kappa *A}{d}*\Delta T = - C*\frac{\kappa *A}{d}*\left( {37 - T_{Amb} } \right) $$

where κ and d are the thermal conductivity and thickness of the box insulation respectively, A is the total box internal surface area, T_Amb_ is the ambient temperature in Celsius, and C is a constant depending on the box geometry (for cubic shaped boxes, the constants are similar). The duration (t) for the battery pack to maintain the culture temperature would be:

5 $$ t = \frac{{E_{bat} }}{{ - \left( {\frac{dQ}{{dt}}} \right)}} $$

where E_bat_ is the energy of the battery pack.

To estimate the insulation and size of the battery pack, we tested three box insulation/battery configurations at refrigerator temperature (4 °C), as shown in Table 3. We can see that a commonly used Styrofoam box with 4 D-cell batteries can keep the µCRAIT box at the target 37 °C for 9.5 h. However, a single-wall VIP insulated box with 4 D-cell batteries can last 57.5 h, and a dual-wall VIP box with similar insulation thickness but doubled D-cell batteries can double the working time to 114 h (almost 5 days), more than sufficient for the 3 days required by the CBMN sample transportation. Considering the facts that all the box dimensions and insulation thicknesses are similar and the thermal conductivity of Styrofoam is about 4–13 times that of VIP (~ 0.030–0.040 vs. ~ 0.003–0.007 Wm^−1^ K^−1^)^15,16^, the results are consistent with Eqs. (4, 5).

**Table 3:** Duration of µCRAIT box working at fridge temperature (4 °C) for three box configurations.

Insulating box info						# of D-cell alkaline batteries	Duration t (h)
Box name	Insulation material	Total insulation thickness d (in)	Internal dimensions				
			L (in)	W (in)	H (in)		
Styrofoam box	Polystyrene foam	1.8	7.8	6.8	6.5	4	9.5
Credo single-wall	VIP	1.8	7.8	6.8	6	4	57.5
Credo dual-wall	VIP	2	7.8	7.8	7.8	8	114

### Mechanical monitoring during transportation

**Figure 4:**
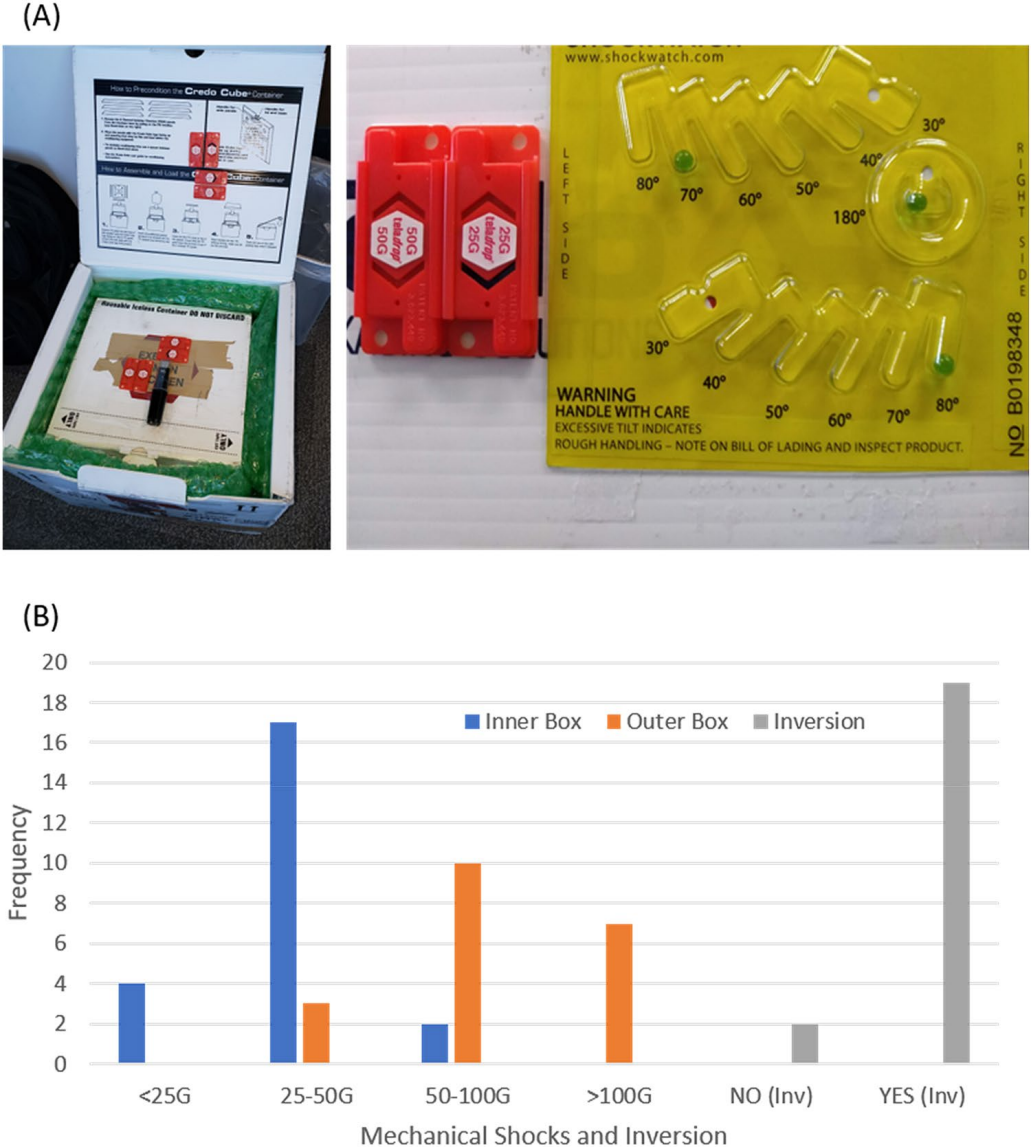
(**A**) Images of a µCRAIT box (with an outer box) and the Drop-N-Tell Shock Indicators and TiltWatch Plus used to monitor the mechanical shock and tilt of the box experienced during transportation; (**B**) mechanical shock G-range and inversion frequency from over twenty shipments between Phoenix, AZ and New York, NY by FedEx Express services.

Mechanical shock is another concern for the µCRAIT box. Mechanical shock during transportation is a threat to electronic products in general^17^, but it is more significant for transporting actively working electronics, such as the µCRAIT box, because damage to the electronics during transportation could ruin all the samples. To monitor the mechanical shock and handling of the box during transportation, we used Teladrop Drop-N-Tell Resettable Shock Indicators to show the G-ranges of the maximum shock (Fig. 4A). The tilt and inversion of the box were monitored by TiltWatch Plus. Over 20 shipments have been conducted between Phoenix, AZ and New York, NY using FedEx Express services (FedEx Corporation, Memphis, TN).

Our initial shipment tests used the commercial VIP Credo box with just a plastic outer. However, we observed damage to the plastic outer as well as the VIP panels over repeated shipments. To avoid box damage and reduce mechanical shock experienced by the µCRAIT box, we housed the µCRAIT box inside another outer box cushioned by bubble wraps (Fig. 4A). 50G and 100G Shock Indicators were attached to the outer box, and 25G and 50G Indicators were attached to the inner box. The G-ranges and frequency experienced by the boxes are shown in Fig. 4B. We can see that the outer box can experience > 100G shocks, and the inner box had shocks mostly in 25–50G range, but can also go above 50G. The TiltWatch Plus showed all boxes were tilted during shipments; over 90% were also turned upside down (Fig. 4B). These results are consistent with FedEx’s policy that orientation of the package is not guaranteed during shipment.

Through the shipping experiments, we found that the PBCs and cable connections were not affected much during transportation. The impact of the mechanical shocks was mainly on the battery holder. Our initial battery holder used metal clips to hold the battery, which can be permanently deformed during transportation to disconnect the battery power. Switching to a battery holder with elastic spring as the battery contact solved this issue. Momentary disconnects of the battery power were still observed during shipment according to our system data log, but it did not affect the system performance due to transient nature of the event.

### CBMN assay using µCRAIT box

To test the µCRAIT box for CBMN assay, non-irradiated and irradiated human blood samples collected at Columbia University’s Center for Radiological Research (New York, NY) were sent to The University of Arizona’s Center for Applied NanoBioscience and Medicine (Phoenix, AZ) by FedEx Overnight inside the 37 °C µCRAIT box, and then shipped back to Columbia University immediately. The controlled temperature condition of the box allowed incubation of cells for the CBMN assay to start immediately following sample draw, gamma-irradiation of blood, as well as during transportation. A set of replicating control samples was also cultured in a laboratory 5% CO_2_ incubator for comparison. As suggested by IAEA^4^, for cell culture without 5% CO_2_, sample tubes should be fully capped, which was the case for our µCRAIT box samples. This will also avoid any sample leakage during transportation. For the laboratory CO_2_ incubator, sample tubes were loosely capped to allow gas exchange. We did observe a decrease of mitotic index without CO_2_ (see Supplemental Figure S2). However, this decrease is not significant enough to prevent CBMN assay in our current setting. The data of accelerated RABiT-II CBMN assay^8^ of the control blood samples and samples after 2-days of shipping in µCRAIT box were obtained by processing all the samples in one 96-well plate at the same time.

**Figure 5:**
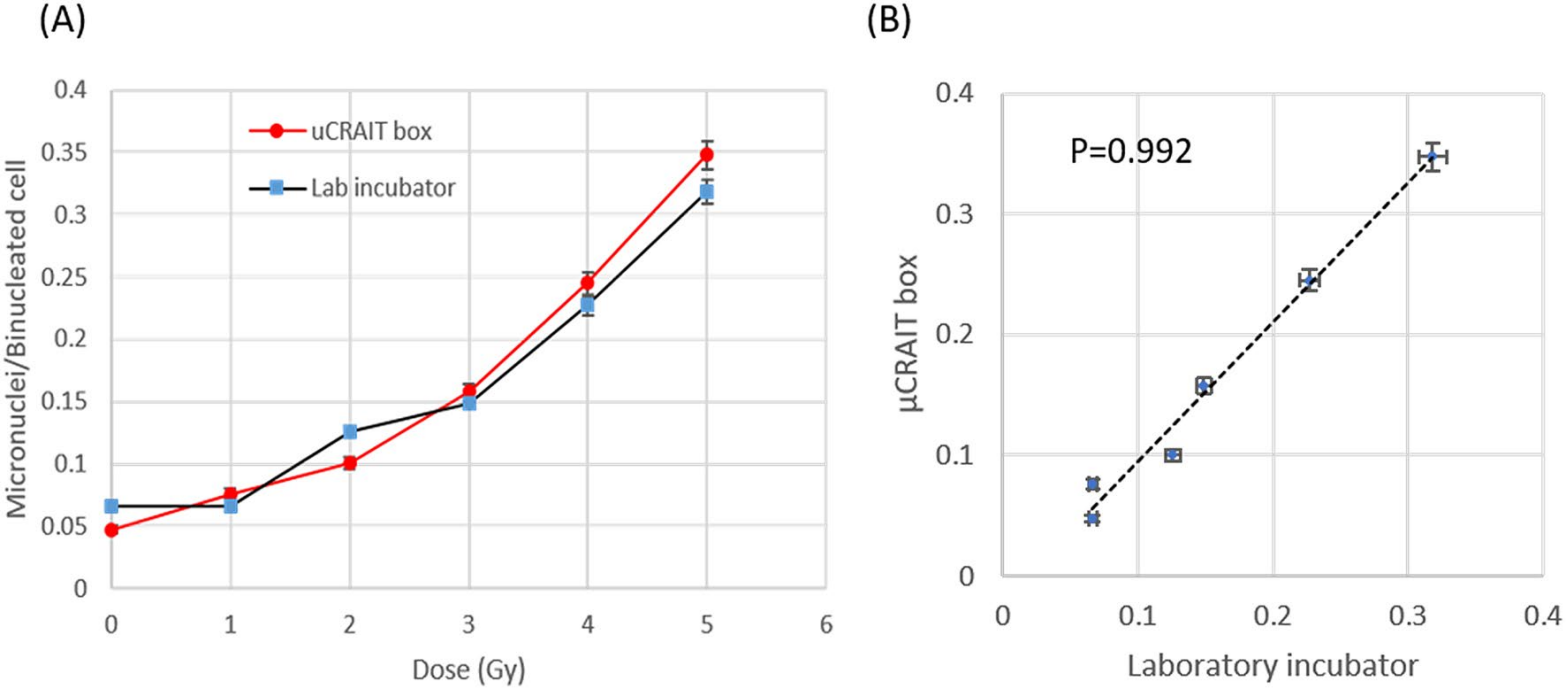
(**A**) Dose curves of CBMN assays generated by cell cultures inside a µCRAIT box during transportation and a laboratory CO_2_ incubator; both show clearly dose response; (**B**) dose responses from µCRAIT box and the control laboratory incubator have a high linear correlation with a Pearson’s correlation coefficient of 0.992; a linear trendline is also shown.

The dose curves of micronuclei yield in binucleated cells in samples cultured in the µCRAIT box and cultured in laboratory incubator are shown in Fig. 5A. Clear dose-dependent curves have been observed for both conditions. A highly linear relationship exists between the two data sets with a Pearson’s correlation coefficient of 0.992 and a 95% confidence interval of (0.921, 0.999) (Fig. 5B). These data clearly demonstrate the feasibility of µCRAIT box for cell culture during transportation for CBMN and possibly other cytogenetic biodosimetry assays.

## Discussion and conclusion

A significant effort of this project is to ensure the cell culture temperature by the µCRAIT box is within 37 ± 0.5 °C. This tight temperature requirement is suggested by IAEA for cytogenetic biodosiemtry assays, such as DCA and CBMN assays^4^. The rationale behind it is that too low a temperature will result in a poor yield of metaphase or binucleated cells. If the temperature is too high, cells will progress more rapidly through the cycle with unacceptably high numbers of second-division metaphase or binucleated cells^9^.

Low cost is also wanted for national stockpile. Commercial shipping incubators with metal construction and rechargeable batteries can cost over $5,000 (iQ5 from MicroQ Technologies). By using alkaline batteries and conventional shipping boxes, the cost for the current µCRAIT box is estimated to be < $400. A large portion of the cost comes from the vacuum insulated Credo shipping box, which cost $310/box. The current µCRAIT box is housing only one 96-tube rack. By scaling up the box size, more sample racks can be housed inside the µCRAIT box and we expect the cost of the box per sample rack would drop accordingly because box volume scales faster than the surface area. If the box cost drops to $100/rack, the cost for national stockpile for 1 million people in a metropolitan area would cost ~ $1 million. At this time, a clear guidance is still needed from policy makers regarding the cost, but we feel this cost is feasible within the total budget of local/Federal government (e.g., ~ $12B for the State of Arizona in Fiscal Year 2021). Furthermore, a significant cost source of national stockpile can come from warehouse storage. Our µCRAIT box has the potential to be folded during storage, which can also lower the cost.

There are still areas that the µCRAIT box can be further developed. For example, a simple resistive heating was used for the µCRAIT box because we expect the box ambient temperature to be < 37 °C during transportation in most cases. There are areas in the U.S. and around the world where the environmental temperature can be > 37 °C during the summer. However, the box could still experience an ambient temperature of < 37 °C during transportation if it is housed inside a temperature regulated vehicle or building. For the rare scenario of > 37 °C ambient temperature, we are also developing a µCRAIT box with a passive phase change material layer to neutralize the heat influx from outside to maintain the desired box temperature.

Another issue of our existing µCRAIT box is the time it takes to heat samples to the target temperature. Currently heating an empty box from room temperature (~ 25 °C) to a steady state 37 °C takes ~ 2 h. Because the internal space of the box is heated without active convection, it can take even longer (over 10 h) to warm a sample rack from room temperature. To address this issue, we currently preheat the samples in a conventional oven before we put them into a pre-warmed µCRAIT box for CBMN assay. To reduce the heating time, a fan can be integrated in our future µCRAIT box to generate forced convection inside the box. Our preliminary results have shown that intermittent operation of a mini-fan can dramatically decrease the heating time with increased temperature uniformity inside the box.

As we mentioned before, current PCBs do not touch at their edges to form an enclosed isothermal surface, which can cause the internal box temperature lower than that of the boards. We envision our future µCRAIT box would have a fully enclosed heat-conductive isothermal surface to address this issue. To demonstrate the concept of the µCRAIT box, our current box was designed to house just one 96-tube rack. We are also in the process of scaling up the box to host more sample racks.

In summary, we have demonstrated a novel transportation box (µCRAIT) for disruptive cytogenetic biodosimetry logistics that uses traditional insulated shipping box, microcontrolled simple resistive heating, low cost and long shelf life alkaline battery to achieve precise temperature control of the box’s internal space at 37 °C. The temperatures of the isothermal PCB surfaces can be controlled within 0.01 °C of the setpoint with standard deviations of < 0.04 °C. After box calibration, the temperature is very uniform in the center of the box, and a dummy 96-tube rack payload showed a steady state temperature variation of < 0.1 °C across the payload. With 8 D-cell alkaline batteries, the box can maintain 37 °C at 4 °C ambient temperature for almost 5 days, long enough for the cell culture of cytogenetic biodosimetry assays. The mechanical shock and tilt of the box during transportation by a commercial shipping company (FedEx) were also characterized, and an appropriate battery holder was selected to address the permanent battery disconnect issue. Finally, successful biodosiemtry CBMN assay was demonstrated using blood samples cultured by the µCRAIT box during transportation with a clear dose-dependent curve of micronuclei per binucleated cell ratio that has high linear-correlation with the laboratory control samples. Currently, civilian radiation countermeasure response in the U.S. is still planned at local government level^18^. The U.S. Department of Defense (DOD) has established a biodosimetry network for military personnel based on a group of biodosimetry methods, including LDK, traditional cytogenetic assays (DCA and premature chromosome condensation (PCC)), and electron paramagnetic resonance (EPR)^19^. High throughput biodosimetry methods such as the one reported in this manuscript are still highly needed. With the demonstrated feasibility of sample pre-processing during transportation to dramatically reduce the response time, the µCRAIT box could have significant impact on existing biodosimetry response for radiation countermeasures in several aspects. The box is designed for any cellular biodosimetry assay which requires significant cell culturing time. Thus, it can potentially be used for the two commonest cellular biodosimetry assays, specifically CBMN assay, as described here, and the DCA assay^20^, both of which require significant cell culturing time. A third commonly used cellular biodosimetry assay, γ-H2AX, does not generally require cell culturing; however, if the γ-H2AX assay is being used as an assay for residual non-repaired DNA damage^21^, then the µCRAIT box would potentially be useful. Finally, besides biodosimetry, the box also has potential impacts on other applications such as biobanking, novel molecular or cell-based assays or therapies^10,11,22^.

## Materials and methods

### Ethics statement

All blood collections were approved by the institutional review boards of Columbia University (IRB# AAAF2671) and done after written informed consents were given. All experiments were performed in accordance with the relevant guidelines and regulations.

### µCRAIT box design and prototyping

PCBs with 1.5-mm-thick Fire Retardant 4 (FR4) substrate and two or four layers of 35-µm-thick copper were used to build the microcontroller. The PCB layout was designed in house and fabricated by a local vendor (Advanced Circuits, Chandler, AZ). The schematics of the Wheatstone bridge and heater circuits can be seen in Figure S1. The design left most copper on the board for good thermal conduction. The system was also assembled in house. Ribbon cables were used to connect the PCBs together. The frame holding the PCBs was printed by an uPrint SE Plus 3D printer (Stratasys, MN). The VIP insulating boxes were purchased from Pelican BioThermal, LLC (Plymouth, MN).

### Temperature calibration and characterization

The thermistors for PCB temperature sensing were purchased from Vishay Americas, Inc (Part# NTHS1206N02N1002JE) with 10 kΩ at 25 °C. Three precision resistors (10 kΩ, 0.1% tolerance TNPW120610K0BEEN by Vishay/Dale) were used as the known resistors in the Wheatstone bridge. The sextic polynomial was fitted by Microsoft Excel. The RTD reference thermometer (SKU: THS-222-555) was purchased from ThermoWorks (American Fork, UT). The laboratory incubator for temperature calibration was a VWR 1545 General Purpose Incubator. The four wired thermistors were purchased from TE Technology, Inc (MP-2444). They were mounted on the stick by heat shrink tubing and controlled by a customer Wheatstone-bridge controller board for temperature measurement. The 96-tube rack format 2D-barcoded Matrix 1.0 ml sample storage tubes were purchased from ThermoFisher Scientific Inc (item# 3850). The tube caps were the SepraSeal Caps from the same company (Item#4464).

### Battery selection and capacity

D-cell alkaline batteries under different brand-names (Duracell, Energizer and Wincell) were purchased from local stores. Their capacities were characterized by their endurance to power an electronic load under constant power (0.2 W), which has shown to be similar with a coefficient of variation (CV) of 5%. The coin cell battery was the CR2032 battery. The Styrofoam box was one of the common insulating boxes for shipping cold-chain reagents that we found in our lab. The single-wall and dual-wall VIP Credo boxes were purchased from Pelican BioThermal, LLC (Plymouth, MN).

### Mechanical monitoring during transportation

The 25G, 50G and 100G Teladrop Drop-N-Tell Resettable Shock Indicators were purchased from Telatemp Corp (Anaheim, CA). The TiltWatch Plus was purchased from Uline (Pleasant Prairie, WI). The metal-clip and the spring-loaded battery holders were both purchased from Digi-Key Electronics (Part# 36-186-ND, and 708-1420-ND).

### CBMN assay using µCRAIT box

Blood samples (2 ml) were collected into heparinized vacutainer tubes (BD, Franklin Lakes, NJ) from 4 healthy volunteers at the Columbia Center for Radiological Research (New York, NY). Blood samples were exposed to 0 (control), 1.0, 2.0, 3.0, 4.0 or 5.0 Gy of γ-rays at a dose rate of 0.70 Gy/min at Gammacell 40 ^137^Cs irradiator (Atomic Energy of Canada Ltd., Mississauga, Canada). 60 μl of human blood samples were pipetted into 1 ml 2D-barcoded Matrix storage tubes (Thermo Fisher Scientific Inc., Waltham, MA) and 500 μl of PB-MAX karyotyping media (Thermo Fisher Scientific) was added. Two identical sets of Matrix tubes with irradiated and non-irradiated blood samples were put into two ANSI/SLAS microplate format compatible 96-tube racks (24 tubes in each rack). One rack with samples was placed into the laboratory incubator at 37 °C under 5% CO2 atmosphere with the tube tops loosely capped. The second sample rack was prewarmed to 37 °C for 30 min inside an incubator with the tube tops fully capped by the SepraSeal caps, and then loaded into prewarmed µCRAIT box for shipment. The box was shipped to The University of Arizona (Phoenix, AZ), and then shipped back to Columbia University (New York, NY) the same day via FedEx Overnight. On the second day after blood draw the µCRAIT box with blood samples was successfully delivered to the Columbia Center for Radiological Research. After 44 h of cell incubation, cytochalasin-B (Sigma-Aldrich, St. Louis, MO) was added to all tubes with samples to block cytokinesis of proliferating lymphocytes at a final concentration of 6 μg/ml and cells were cultured for an additional 10 h (total incubation time—54 h) in CO_2_ incubator and µCRAIT box respectively. After completion of cell culture, the samples were collected into one 96-well plate (two wells were filled with 250 μl of samples from each Matrix tube) and processed for accelerated RABiT-II CBMN assay as described before^8^. The dose curve plots, linear trendline, and Pearson’s correlation coefficient with 95% confidence interval were done by Microsoft Excel and R software.

## Supplementary Information

Supplementary Information.
