# Supplementary material for: Transportation container for pre-processing cytogenetic assays in radiation accidents
Source: Sci Rep. 2021 May 17;11:10398. doi: 10.1038/s41598-021-89832-x (PMC8129553; doi:10.1038/s41598-021-89832-x)
Supplement: Supplementary file 1 — Supplementary Information. [file 41598_2021_89832_MOESM1_ESM.docx]

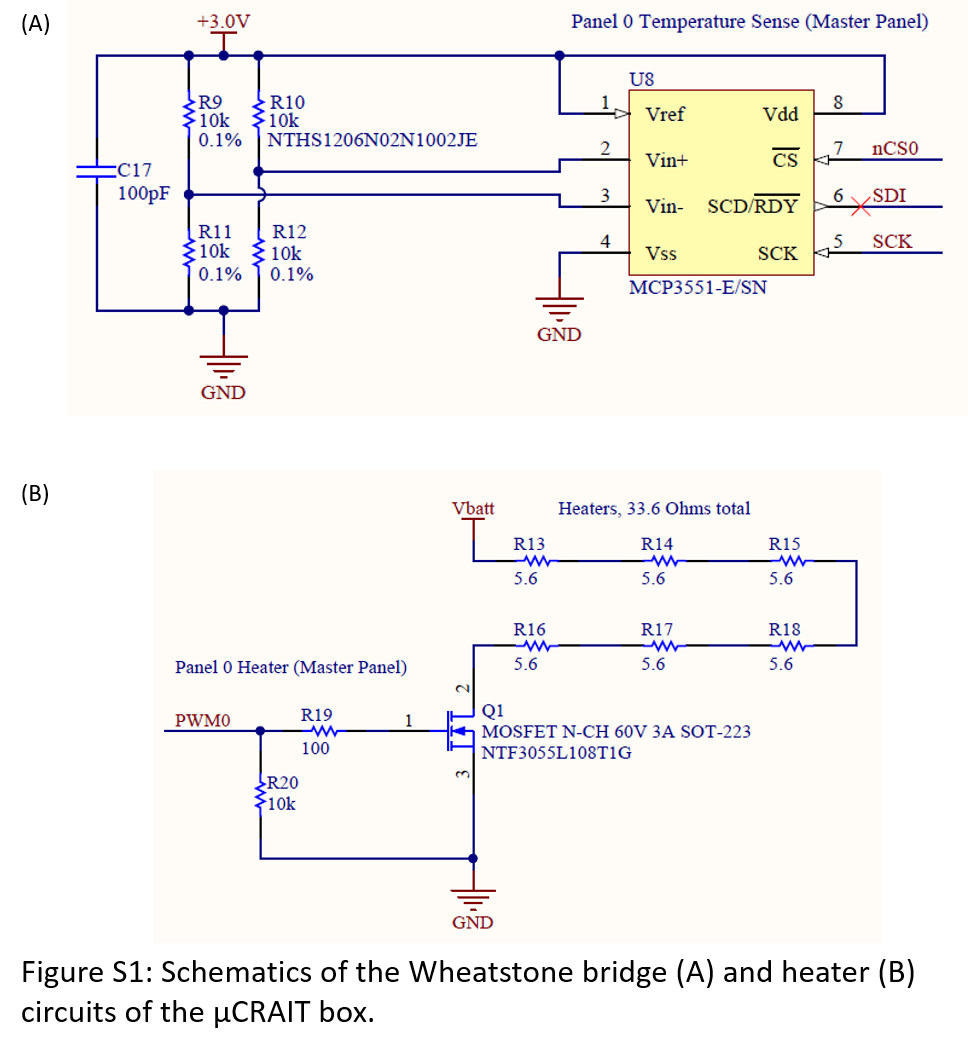


**Figure S1:** Schematics of the Wheatstone bridge (A) and the heater (B) circuits of the µCRAIT box.

**Figure S2:** Logarithm of the average numbers of binucleated cells (BN) from the four donors vs. radiation dose for both the CO_2_ incubator and the µCRAIT box. Linear regression by R software showed significant difference between the two model intercepts (p=0.00268, with a lower intercept for the µCRAIT box), but not between the slopes (p=0.62421).
